# Computer-aided anatomy recognition in intrathoracic and -abdominal surgery: a systematic review

**DOI:** 10.1007/s00464-022-09421-5

**Published:** 2022-08-04

**Authors:** R. B. den Boer, C. de Jongh, W. T. E. Huijbers, T. J. M. Jaspers, J. P. W. Pluim, R. van Hillegersberg, M. Van Eijnatten, J. P. Ruurda

**Affiliations:** 1 Department of Surgery, University Medical Center Utrecht, Heidelberglaan 100, 3584 CX Utrecht, The Netherlands; 2 Department of Biomedical Engineering, Eindhoven University of Technology, Groene Loper 3, 5612 AE Eindhoven, The Netherlands

**Keywords:** Minimally invasive surgery, Artificial intelligence, Anatomy recognition, Computer vision

## Abstract

**Background:**

Minimally invasive surgery is complex and associated with substantial learning curves. Computer-aided anatomy recognition, such as artificial intelligence-based algorithms, may improve anatomical orientation, prevent tissue injury, and improve learning curves. The study objective was to provide a comprehensive overview of current literature on the accuracy of anatomy recognition algorithms in intrathoracic and -abdominal surgery.

**Methods:**

This systematic review is reported according to the Preferred Reporting Items for Systematic Reviews and Meta-Analyses (PRISMA) guideline. Pubmed, Embase, and IEEE Xplore were searched for original studies up until January 2022 on computer-aided anatomy recognition, without requiring intraoperative imaging or calibration equipment. Extracted features included surgical procedure, study population and design, algorithm type, pre-training methods, pre- and post-processing methods, data augmentation, anatomy annotation, training data, testing data, model validation strategy, goal of the algorithm, target anatomical structure, accuracy, and inference time.

**Results:**

After full-text screening, 23 out of 7124 articles were included. Included studies showed a wide diversity, with six possible recognition tasks in 15 different surgical procedures, and 14 different accuracy measures used. Risk of bias in the included studies was high, especially regarding patient selection and annotation of the reference standard. Dice and intersection over union (IoU) scores of the algorithms ranged from 0.50 to 0.98 and from 74 to 98%, respectively, for various anatomy recognition tasks. High-accuracy algorithms were typically trained using larger datasets annotated by expert surgeons and focused on less-complex anatomy. Some of the high-accuracy algorithms were developed using pre-training and data augmentation.

**Conclusions:**

The accuracy of included anatomy recognition algorithms varied substantially, ranging from moderate to good. Solid comparison between algorithms was complicated by the wide variety of applied methodology, target anatomical structures, and reported accuracy measures. Computer-aided intraoperative anatomy recognition is an upcoming research discipline, but still at its infancy. Larger datasets and methodological guidelines are required to improve accuracy and clinical applicability in future research.

Trial registration: PROSPERO registration number: CRD42021264226

**Supplementary Information:**

The online version contains supplementary material available at 10.1007/s00464-022-09421-5.

Minimally invasive surgery (MIS) reduces surgical trauma by enabling surgery through small incisions rather than a large wound as in open surgery. Most minimally invasive procedures are highly complex, and have substantial learning curves and significant complication rates [1–3]. The zoomed-in surgical view during MIS is valuable for detailed surgical dissection, but also poses a challenge as it limits a broad surgical overview of the operating field for proper anatomical orientation, especially for novice surgeons. Combined with the presence of vital structures in the operating field, this might result in injury of important anatomical structures and complications. The technology enabling MIS also provides an interface during surgery, which can function as a medium for video analysis algorithms. Such algorithms may improve surgical training methods, increase anatomical orientation and prevent tissue injury, thereby possibly improving perioperative outcomes and potentially reducing learning curves.

Video analysis algorithms can be divided into model-based and data-based algorithms. In model-based algorithms, all assumptions about the problem are made explicit in the form of a model based on pre-specified fixed rules. In data-based algorithms, the features of anatomical structures are not pre-specified, but learned from the data itself. Data-driven algorithms include methods in the realm of artificial intelligence (AI), which refers to the use of computer algorithms to simulate independent human-like reasoning. Machine learning is a subtype of AI in which algorithms learn to perform tasks from data and improve through experience [4]. Deep learning is in turn a subtype of machine learning and includes algorithms comprising multiple “deep” layers of connected neurons to improve model predictions for complex tasks [4]. Deep learning algorithms for anatomical recognition are commonly trained using a large dataset of surgical video frames with anatomical structure(s), manually labeled by surgical experts.

Research regarding the application of deep learning in surgical videos has increased over the latest years. The fast and precise analysis of images deep learning provides, has already proven valuable in multiple medical disciplines, such as detection tasks in radiology, classification of skin cancer in dermatology, classification of fundus photographs in ophthalmology, and recognition of polyps during colonoscopy [5–8]. However, deep learning algorithms often require large annotated datasets, which complicates the development of these algorithms as such datasets are frequently unavailable. Another hurdle in the development relates to the ‘black box’ principle of complex AI algorithms [9]. The process and working of the algorithms cannot be inspected, which reduces explainability of the predictions of the algorithms.

Image analysis of surgical videos has its own challenges. Recognition of anatomy can be hindered by soft and deformable tissue nature, intraoperative tissue manipulation by the surgeon, the surgical dissection itself, resulting in differences in anatomy as operative steps are consecutively performed, and tissue movement due to breathing, heartbeat, arterial pulsations, and patient positioning. Despite these challenges, computer-aided anatomy recognition has the potential to improve the surgeon’s orientation during operations, reduce tissue injury and decrease learning curves for MIS, and its added value should therefore be explored.

Publications regarding computer-aided anatomy recognition in surgical videos increased substantially in latest years, most commonly using videos of laparoscopic cholecystectomy [10, 11]. A previous systematic review on deep learning visual analysis in laparoscopic surgery reported on algorithms with a wide range of purposes, including prediction of operating time and surgical phase recognition [12]. In this previous review, a detailed clinical and technical overview of specifically anatomy recognition algorithms is missing. Moreover, structured methodological recommendations on how to develop successful surgical video analysis algorithms for computer-aided anatomy recognition are not available. This review provides an in-depth summary on the current clinical and technical possibilities (and limitations) of computer-aided anatomy recognition, and recommends standardized methodology for future studies. This is important to facilitate high-quality future studies in this relatively new field of research. This study’s objective was to provide a comprehensive overview of current literature on the accuracy of anatomy recognition algorithms in intrathoracic and -abdominal surgery. This can stimulate the development of high-quality anatomy recognition algorithms, which may improve surgical training and patient safety during surgery.

## Materials and methods

### Protocol and registration

This systematic review is reported according to the Preferred Reporting Items for Systematic Reviews and Meta-analyses (PRISMA) guidelines [13]. The predefined study protocol was registered in the international PROSPERO-registry for systematic reviews under registration number CRD42021264226. A systematic literature search was conducted in Pubmed, Embase and IEEE Xplore databases and updated up to January 4th 2022.

### Eligibility criteria

The inclusion criteria consisted of original articles reporting on anatomy recognition in intrathoracic or -abdominal surgery using a laparoscopic or robot-assisted approach in English language, conducted in human patients. The following exclusion criteria were applied: no anatomy recognition performed, requirement of additional intraoperative calibration equipment or imaging modalities, review articles, and no full-text available.

### Information sources and search

Terms that were included in the search were “recognition”, “surgery”, “artificial intelligence”, and their synonyms, followed by the lungs, esophagus, all relevant intra-abdominal organs, and their related surgical procedures. The complete search strategy is provided in Table 1.

**Table 1:** Complete search strategy

#1 AND	#2 AND	#3 AND	#4
Recognition Anatomic landmark Segmentation Detection Annotation Registration Classification Delineation Deformation	Surgery Laparoscop* Thoracoscop* Surgical* Intraoperative Operation	Artificial intelligence Deep neural network Deep learning Convolutional neural network CNN Machine learning Algorithm Augmented realit* Mixed realit* Surgical navigation	Lung Pulmona* Lobectom* Trachea* Pneum* Bronch* Upper-GI Esophag* Oesophag* Abdominal Gastric Gastrectomy Stomach Bowel Duoden* Intestin* Jejun* Ileum Colon* Colectomy Appendix Appendectomy Colorectal Rectal Rectum HPB Liver Hepatectomy Hepatic Pancrea* Galbladder Cholecystectomy Spleen Splenic Uterus Ovar* Hysterectomy Fallopian tube Kidney Nephrectomy Ureter Bladder Cystectomy Prostat*
**MeSH-Terms**s Anatomic landmarks	**MeSH-Terms** Laparoscopy Thoracoscopy Surgery, Computer-Assisted/methods Robotic surgical procedures	**MeSH-Terms** Augmented reality Algorithms Artificial intelligence Image Processing, Computer Assisted/methods	**MeSH-Terms** Lung Lung neoplasms/surgery Esophagectomy Gastrectomy Colectomy Hepatectomy Pancreatectomy Cholecystectomy Hysterectomy Nephrectomy Cystectomy Prostatectomy
**Entree-Terms** Anatomic landmark/exp	**Entree-Terms** Laparoscopy/exp Thoracoscopy/exp Surgery/exp	**Entree-Terms** Augmented reality/exp Algorithm/exp Artificial intelligence/exp Deep learning/exp Image processing/exp	**Entree-Terms** Lung/exp Esophagus resection/exp Gastrectomy/exp Colon resection/exp Intestine resection/exp Liver resection/exp Pancreatectomy/exp Cholecystectomy/exp Hysterectomy/exp Nephrectomy/exp Cystectomy/exp Prostatectomy/exp

*A wildcard symbol that broadens the search by finding words that start with the same letters

### Study selection

After removal of duplicates, articles were screened on title and abstract independently by two researchers (WH and RdB) according to the inclusion and exclusion criteria. Subsequently, additional articles were sought by cross-referencing of the included articles. If an article was selected by only one researcher, consensus was reached whether to include this article or not. The same method was applied to the full-text review.

### Data collection process

The following data were extracted by two researchers (WH and RdB): year of publication, study population and design, surgical procedure, algorithm type, pre-training methods, pre- and post-processing methods, data augmentation, anatomical annotation of the reference standard, number of training and testing data, model validation strategy, goal of the algorithm, target anatomical structure, accuracy scores and inference time.

### Definitions for data extraction

The algorithms described in the included articles were divided into model-based and AI-based algorithms based on machine learning or deep learning. The goal of the algorithm was divided into five groups: segmentation, bounding box detection, edge detection, organ presence recognition, and classification. Segmentation aims at assigning individual pixels to a certain anatomical structure. Bounding box detection indicates the location of an anatomical structure using a rectangular shape. Edge detection indicates boundaries of anatomical structures. Organ presence recognition indicates whether an anatomical structure is present in the frame or not. Classification algorithms aim to allocate anatomical structures to different categories, for instance the degree of vascularity of the target structure. Inference times of the algorithms were extracted from the included articles, which indicates the time it takes for the algorithm to process the data and make a prediction.

### Quality assessment and risk of bias

Risk of bias assessment of all included studies was performed using the Quality Assessment of Diagnostic Accuracy Studies (QUADAS-2) criteria, modified for algorithm-based research [14]. Studies were evaluated on four criteria: patient selection, index test, reference standard, and flow and timing. The methodological quality was assessed by the Joanna Briggs Institute (JBI) critical appraisal checklist, modified for machine learning research [15]. Both assessments were independently conducted by two researchers (WH and RdB) and consensus was reached in case of any disagreements by a consensus meeting.

### Accuracy measures

The primary outcome was accuracy of the anatomy recognition algorithms. The accuracy was defined as the ability to recognize anatomical structures with their correct label, in concordance with the provided reference standard. All provided outcome measures to evaluate accuracy of the algorithms were accepted. Explanations of the accuracy measures are included in Table S1.

### Synthesis of results

Because of the wide variety in the used outcome measures and the lack of standardization on reporting algorithms for anatomy recognition, the included studies are described in a narrative manner without statistical comparative tests, and no meta-analysis could be performed.

## Results

### Study selection

**Fig. 1:**
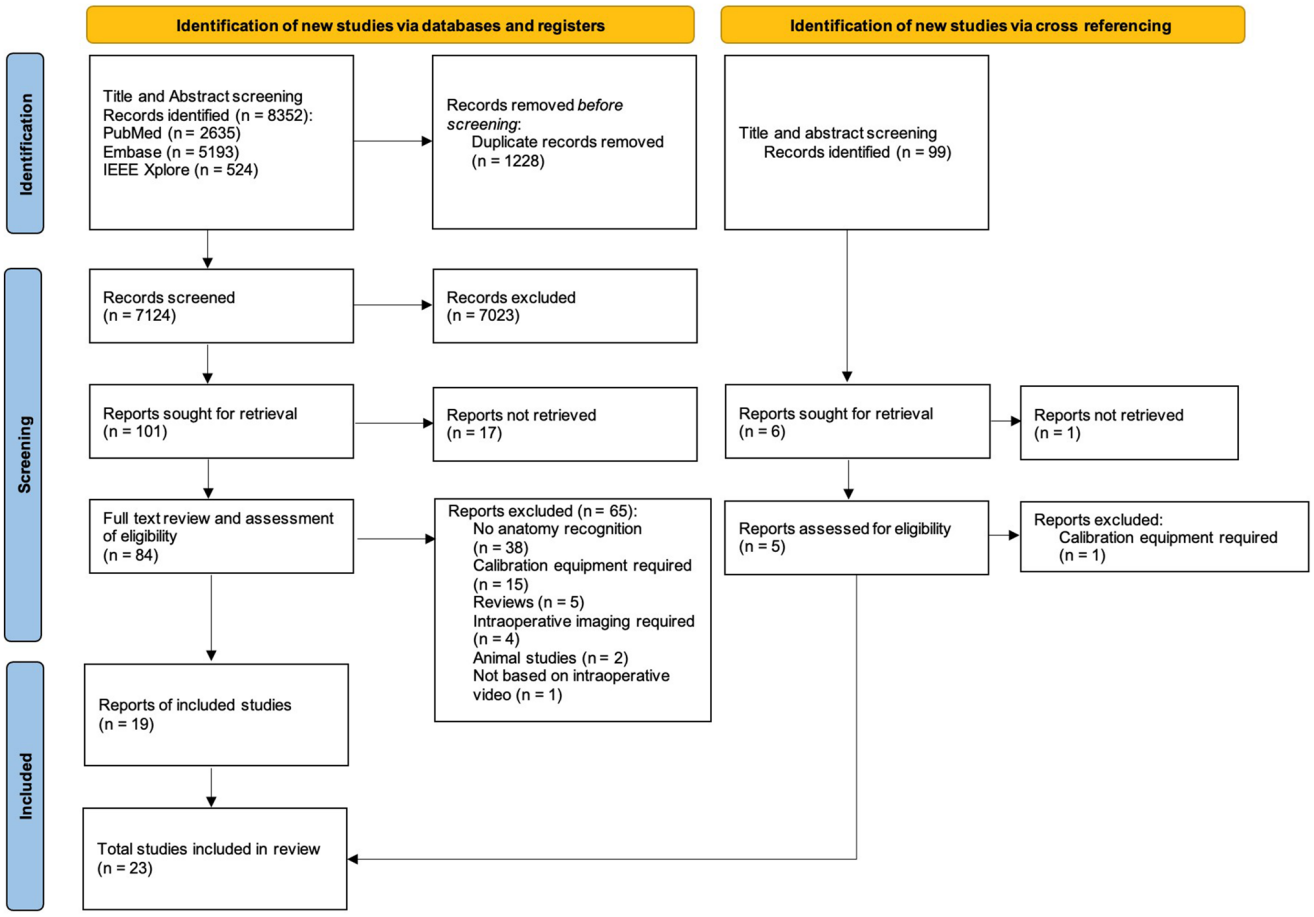
PRISMA flowchart

After removal of 1228 duplicate articles, 7124 studies were identified in the search, of which 7023 were excluded in the title and abstract screening (Fig. 1). After full-text screening, 81 articles were excluded due to no anatomy recognition (*n* = 38), no retrieval of full-text (*n* = 17), requirement of calibration equipment (*n* = 15), reviews (*n* = 5), requirement of intraoperative imaging (*n* = 4), and animal studies (*n* = 2). Four additional articles were included via cross-referencing, resulting in a total of 23 studies.

### Study characteristics

Characteristics and results of the included studies are summarized in Table 2. Year of publication ranged from 2008 until 2021. Fourteen of the 23 studies (61%) were published in 2020 and 2021, and all used AI-based algorithms [10, 11, 16–27]. The majority of studies (*n* = 20; 87%) used a retrospective study design, a prospective or mixed design was used in three studies [20, 23, 28]. The most frequently reported procedure was cholecystectomy (*n* = 8; 35%) [10, 11, 16, 19, 23, 26, 27, 29] followed by hysterectomy and other gynecological procedures (*n* = 5; 22%)[20, 24, 30–32], nephrectomy (*n* = 3; 13%) [25, 28, 33], abdominal laparoscopy (exact procedure not specified; *n* = 3; 13%) [17, 34, 35], hepatectomy (*n* = 1; 4%) [36], lung cancer resection (*n* = 1; 4%) [21], robot-assisted gastrectomy (*n* = 1; 4%) [18], and transanal total mesorectal excision (TATME) (*n* = 1; 4%)[22]. Five studies (22%) analyzed robot-assisted procedures [18, 25, 28, 33, 36], 18 studies (78%) used laparoscopic or thoracoscopic procedures [10, 11, 16, 17, 19–24, 26, 27, 29–32, 34, 35].

**Table 2:** Details of included studies

Author (year)	Surgical procedure	Population	Algorithm type	Model used	Annotation of the reference standard	Training data	Testing data	Goal of the algorithm	Target anatomical structure	Accuracy		
										Dice	IoU	Other
Akbari et al. (2008) [34]	Abdominal laparoscopy	2 patients	Model-based	Change detection algorithm	1 medical doctor	N/A	20 image pairs	Segmentation	Renal and gastric vasculature	NR	NR	Sensitivity: 0.95 Specificity: 0.92
Akbari et al. (2009) [29]	Lap. cholecystectomy	19 patients	Model-based	Fourier transform	NR	N/A	80 sequential image pairs	Segmentation	Cystic artery	NR	NR	Sensitivity: 0.95 Specificity 0.96
Artemchuk et al. (2010) [35]	Abdominal laparoscopy	1 patient	Machine learning	Neural network	NR	100 frames	100 frames	Organ presence recognition	Gallbladder + Liver	NR	NR	Precision: 0.86
Chhatkuli et al. (2014) [31]	Lap. gynaecological procedure	15 patients	Machine learning	SVM	NR	45 frames	75 frames	Segmentation	Uterus	0.80	NR	NR
Prokopetc et al. (2015) [32]	Lap. gynaecological procedure	38 patients	Machine learning	Sparse linear SVM	NR	167 frames	28 frames	Bounding box	Uterus	NR	NR	FPR: 0.21 Recall: 0.95
Amir-Khalili et al. (2015) [33]	Robot-assisted nephrectomy	15 patients	Model-based	Phase change algorithm	1 junior surgeon and 1 expert surgeon	N/A	15 patients	Segmentation	Kidney, tumor/cyst, AA, IVC, RA, RV, and accessory vessels	0.50 (SD: 0.24)	NR	AUC: 0.72 (SD: 0.05)
Haouchine et al. (2016) [36]	Robot-assisted hepatectomy	3 patients	Machine learning	Custom algorithm	NR	N/A	3 patients	Segmentation	Liver	0.81	NR	NR
Nosrati et al. (2016) [28]	Robot-assisted nephrectomy	15 patients	Machine learning	Random forest + pulsation analysis	1 graduate student and 1 junior urologist	N/A	15 clinical cases	Segmentation + alignment	Kidney	0.70	NR	Accuracy: 0.88 FPR: 0.07
Sato et al. (2019) [30]	Lap. hysterectomy	19 patients	Machine learning	OpenCV	NR	NR	NR	Segmentation	Ureter	NR	NR	NR
Nitta et al. (2020) [21]	Thoracoscopic lung cancer resection	25 patients	Deep learning	U-net	Researchers	1890 frames	12 frames	Segmentation	Lung	NR	98%	NR
Tokuyasu et al. (2020) [23]	Lap. cholecystectomy	99 patients	Deep learning	YOLO v3	2 expert surgeons	2339 frames	23 videos	Bounding box	Common bile duct	NR	NR	Precision: 0.32
Mascagni et al. (2020) [27]	Lap. cholecystectomy	201 patients	Deep learning	Deeplab v3 + Xception 65	3 surgeons (training/junior/senior)	1712 frames	571 frames	Segmentation	Gallbladder	NR	89% (SD: 0.9)	NR
Loukas et al. (2020) [19]	Lap. cholecystectomy	41 patients	Machine learning	VBGMM	1 expert surgeon	241 frames	241 frames	Segmentation	Gallbladder	NR	NR	Accuracy: 0.79 (SD: 0.02)
Zadeh et al. (2020) [24]	Lap. hysterectomy	8 patients	Deep learning	Mask R-CNN	1 junior surgeon and 1 expert surgeon	361 frames	100 frames	Segmentation	Uterus	NR	85%	Recall: 0.97 Precision: 0.99
Sheilkl et al. (2020) [16]	Lap. cholecystectomy	12 patients	Deep learning	TernausNet-11 with a trainable encoder and the SJ loss function.	Medical students	126 frames	42 frames	Segmentation	Liver	NR	74%	NR
Madani et al. (2020) [10]	Lap. cholecystectomy	209 patients	Deep learning	PSPnet: (CNN; ResNet50)	4 expert surgeons	2364 frames	263 frames	Segmentation	Liver	0.92 (SD: 0.10)	86% (SD:12)	Sensitivity: 0.93 (0.10) Specificity: 0.96 (0.04)
François et al. (2020) [20]	Lap. gynecological procedures	79 patients	Deep learning	U-Net (CEBiPα-TiP)	1 surgeon	2749 frames	496 frames	Edge detection	Uterus	NR	NR	Reprojection error: 62.44 pixels
Casella et al. (2021) [25]	Robot-assisted nephrectomy	8 patients	Deep learning	3D FCNN + U-net	1 expert surgeon	1391 frames	240 frames	Segmentation	Renal artery	0.72 (SE: 0.09)	NR	Recall: 0.51(se: 0.14) Precision: 0.90 (SE: 0.11)
Kitaguchi et al. (2021) [22]	Lap. TATME	17 patients	Deep learning	Deeplab v3	2 expert surgeons	400 frames	100 frames	Segmentation	Prostate	0.71 (SD: 0.04)	NR	NR
Loukas et al. (2021) [11]	Lap. cholecystectomy	53 patients	Deep learning	v-pResNet, AvgC	2 expert surgeons	480 frames	160 frames	Classification	Vascularity of the gallbladder wall	NR	NR	AUC: 0.95 Precision: 0.91
Caballas et al. (2021) [26]	Lap. cholecystectomy	1 patient	Deep learning	YOLACT++	NR	321 frames	107 frames	Segmentation	Gallbladder	NR	NR	Average precision: 0.89
Bamba et al. (2021) [17]	Abdominal laparoscopy	9 patients	Deep learning	IBM visual insights	Researchers validated by experts in the field	1070 frames	200 frames	Segmentation	Gastrointestinal tract	NR	NR	Precision 0.91 (95% CI: 0.88 -0.94) Recall: 0.93 (95% CI 0.90 – 0.96)
Kumazu et al. (2021) [18]	Robot-assisted gastrectomy	33 patients	Deep learning	U-Net	2 expert surgeons	1800 frames	80 frames	Segmentation	Connective tissue fibers	0.55	NR	Recall: 0.61

*AA* Abdominal Aorta; *AUC* Area Under receiver operating characteristic Curve; *CI* Confidence Interval; *CNN* Convolutional Neural Network; *FCNN* Fully Convolutional Neural Network; *FPR* False Positive Rate; *IBM* International Business Machine Corperation; *IoU* Intersection over Union; *IVC* Inferior Vena Cava; *Lap* laparoscopic; *N*/*A* Not Applicable; *NR* Not Reported; *RA* Renal Artery; *RV* Renal Vein; *ResNet* Residual Neural Network; *SD* Standard Deviation; *SE* Standard Error; *SVM* Support-Vector Machine; *TATME* Transanal Total Mesorectal Excision; *VBGMM* Variational Bayesian Gaussian Mixture Modeling; *YOLACT* You Only Look At Coefficien Ts; *YOLO* You Only Look Once

A wide variety in the number of patients and frames was observed in the described training datasets, ranging from one patient with 100 frames to 209 patients with a total of 2364 frames [10, 35]. Training datasets are only required for AI-based algorithms, so five studies (22%) with model-based algorithms had no need for a training dataset [28, 29, 33, 34, 36]. One study only used surgical videos and images acquired via a search engine [32].

#### Algorithm type

Of the 18 AI-based algorithms, 13 studies (72%) used a deep learning algorithm [10, 11, 16–18, 20–27]. Four studies based their model on U-net and developed a variation [18, 20, 21, 25]. A total of five model-based algorithms were found [28, 29, 33, 34, 36]. Of these algorithms, four used a phase change detection algorithm that localizes blood vessels based on their pulsation [28, 29, 33, 34]. In addition to the phase change algorithm, one article also applied a random decision forest to learn visual patterns of tissue types and combined these algorithms to align preoperative patient algorithms with the intraoperative laparoscopic images [28]. The remaining model-based algorithm made anatomy segmentations based on structures from a point cloud, a three-dimensional shape reconstruction from stereoscopic images [36].

#### Anatomical annotation of the reference standard

The annotator of the reference standard was mentioned in 16 studies (70%) [10, 11, 16–25, 27–29, 34]. Of those 16 studies, 12 studies (75%) used expert surgeons to annotate the anatomy on the frames [10, 11, 17–20, 22–25, 27, 28]. Concordance via a third annotator or mentioning of the inter-annotator differences was done in five studies (31%) [10, 17, 18, 23, 27]. In the four remaining studies (25%), annotation was provided by either non-medical researchers, medical students, or a medical doctor [16, 21, 28, 34].

#### Pre- and post-processing

Pre- and post-processing are commonly used to increase the accuracy of anatomy recognition algorithms. Pre-processing is applied to make input frames more uniform to simplify the recognition process. Post-processing is used to increase visibility and improve comprehensibility of the output frames [7]. Data pre-processing was performed in 10 studies (43%) [11, 19, 21–25, 29, 32, 34]. Resizing of frames was done in three studies (13%) [22, 24, 25]. In two studies (9%) on change detection algorithms, image registration was performed, where multiple frames are aligned in the same coordinate frame [29, 34]. One study (4%) was particularly focused on the effect of data pre-processing, using generative adversarial networks (CycleGan) to improve a deep learning-based lung region segmentation [21]. Apart from post-processing steps like resizing back to the original image size, five studies (22%) implemented additional post-processing steps [11, 19, 29, 31, 34]. One study (4%) included late fusion of output maps generated per patch by their algorithm [11]. Another study implemented a step to correct for over-segmented images, by merging adjacent regions based on their color similarity [19]. Median filter and a filling holes filter are applied by a study to remove small groups of pixels that differ from their adjacent pixels [34]. Post-processing based on connected region analysis was also used in one of the included studies (4%), this eliminates regions that do not touch an image boundary or that are too small [31]. One study applied post-processing to eliminate unwanted tissue movements caused by surgical instruments [29].

#### Pre-training

Pre-training refers to training a deep learning algorithm using an existing (publicly available) dataset to compensate for a lack of training data. Pre-training was performed in six studies (26%) [16, 20, 22–24, 27]. ImageNet, a large publicly available image database, was used in four of these cases (17%) [10, 16, 22, 24, 37]. Additionally, one study (4%) also pre-trained their model on Microsoft COCO and PASCAL VOC [27]. Another study pre-trained their model on a semantic boundary dataset and one study (4%) did not mention on what dataset pre-training was performed [11, 20].

#### Data augmentation

Data augmentation is a technique to increase the amount of data by adding slightly adjusted copies of the existing data to increase robustness of the algorithm. Six studies (26%) applied data augmentations [11, 16, 21–23, 25]. Rotations and horizontal flips were most often applied (*n* = 5; 22%) [11, 16, 22, 23, 25]. Other data augmentation methods included zooming, vertical flipping, shearing, contrast changes, parallel movements, gaussian blur, and affine transformation (Table S2).

#### Validation strategy

Validation is testing of a model on unseen data to give an unbiased estimate of the model’s accuracy. AI-based algorithms were mostly validated through *k*-fold cross validation, where the dataset is split into ‘k’ groups of which one functions as the test set (*n* = 6; 26%) [10, 11, 19, 22, 27, 32]. This process is repeated until every unique group is used once as the test set. Other validation techniques included holdout-cross validation, leave-one-out cross validation, and random sampling. External validation was only performed in one study (4%) [10]. Model-based algorithms were validated through retrospective clinical cases [28, 29, 33, 34, 36].

### Quality assessment

#### QUADAS-2

Evaluating risk of bias using the modified QUADAS-2 tool for algorithm-based research revealed high overall risk of bias (Table 3). Eight studies (35%) had low risk of bias in patient selection (Table 3) [10, 17, 18, 22–24, 27, 32]. Additionally, low risk of bias was scored for the index test in 13 studies (57%) [10, 11, 16, 18–20, 22, 25, 27, 28, 31–33]. Eight studies (35%) performed the annotations of the reference standard with low risk of bias [10, 11, 17, 18, 22–24, 27]. No studies were attributed high risk of bias for the flow and timing criterion. Detailed QUADAS-2 risk of bias sheets for each study with justification for each judgement are available as supplementary file.

**Table 3:**
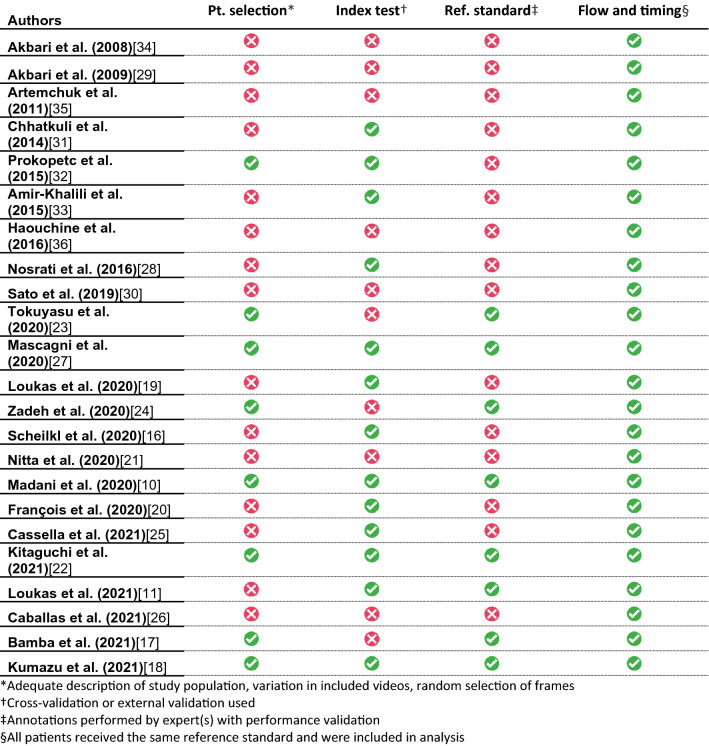
QUADAS-2 tool for risk of bias assessment

#### JBI tool assessment

The quality assessment using the JBI tool adjusted for machine learning is provided in Table 4 and Table 5. A clear objective and description of inclusion criteria of the medical videos was available in 52%. A valid and reproducible data collection and measurement method was reported in 39%. Outcomes were measured in a valid way in 65%. In 87% of the studies, the findings and implications were discussed in detail.

**Table 4:**
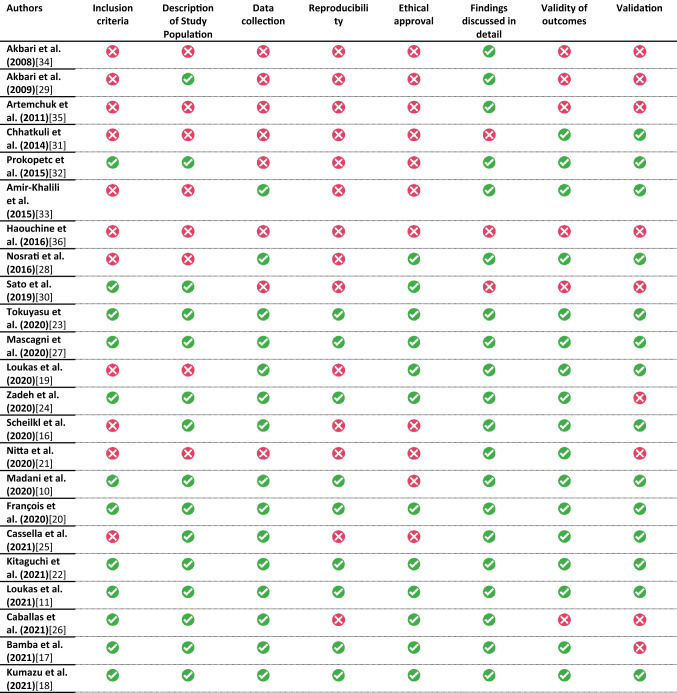
Modified Joanna Briggs Institute (JBI) critical appraisal checklist

**Table 5:** Summarized Modified Joanna Briggs Institute (JBI) critical appraisal checklist

Item	Checklist	Applicability
1	A clear objective and description of inclusion criteria of the medical images/videos	52% (12/23)
2	A detailed description of the study population (how are patients recruited, which operation, laparoscopy/robotic surgery, (dataset))	65% (15/23)
3	A clear description of the data source and how data were collected (method of measurement, machine used, manual annotation, reproducible measurements)	65% (15/23)
4	A valid and reproducible data collection and measurement method	39% (9/23)
5	Attainment of ethical approval?	52% (12/23)
6	Were findings and implications discussed in detail?	87% (20/23)
7	Were the outcomes (performance and result of ML tools) measured in a valid and reliable way?	74% (17/23)
8	Was appropriate cross-validation and evaluation method used?	61% (14/23)

### Accuracy measures

Different accuracy measures were used depending on the objective of the algorithm. Segmentation algorithms were evaluated using Dice, mean average precision (mAP), intersection over union (IoU), area under the receiver operating characteristic curve (AUC), accuracy, precision, recall, sensitivity, specificity, and false positive rate (FPR). In one study, the authors included alternative measures, including one-error, ranking-loss, hamming-loss, and coverage [19].

The detection algorithms included in the present study were evaluated using precision, average precision, recall, and false-positive rate. For edge detection, evaluation measures were reported in pixels, such as the reprojection error or IoU. The only study focusing on classification used accuracy, precision, recall, specificity, and AUC as measures for evaluating the accuracy [11].

In three studies, the model accuracy was tested in a clinical setting [18, 20, 23]. In one study, the authors measured the average duration of marking contours of the uterus for both the surgeons and model [20]. In another study, the model was evaluated with a clinical test whether surgeons agreed with the detected anatomical landmark during laparoscopic cholecystectomy [23]. Lastly, the authors of one study conducted questionnaires for qualitative evaluation of the algorithm’s accuracy by expert surgeons [18].

### Accuracy

A complete overview of the results can be found in Table 2 and Table S3. The included studies demonstrate a wide applicability of anatomy recognition algorithms, varying from organ presence recognition to classification of vascularity of organs and highlighting surgical go- and no-go zones [10, 11, 35]. Included studies used a range of different accuracy measures and only one of the included studies used an external dataset to validate the accuracy, which complicates comparison between studies (Table 2) [10]. Additionally, none of the studies reported on the individual modeling effects of size of the dataset, pre-training, data augmentations, and pre- and post-processing on the accuracy of the algorithm.

The two lowest reported Dice coefficients were 0.50 and 0.55. [18, 33]. However, as larger structures yield higher dice and IoU scores independent of segmentation performance, these studies do not necessarily have the lowest performance. In the study by Amir-Khalili et al. (2015), a model-based approach to recognize vasculature in robot-assisted nephrectomy using a phase change algorithm was used in 15 patients, and annotation of the reference standard was performed by a junior and senior surgeon (Dice coefficient: 0.50) [33]. The study by Kumazu et al. (2021) applied a deep learning algorithm to detect connective tissue fibers in robot-assisted gastrectomy to define safe surgical dissection planes (Dice coefficient: 0.55) [18]. The authors trained this model using 33 surgical procedures, resulting in 1800 frames and annotation was performed by expert surgeons. The use of pre-training, pre- or post-processing, and data augmentation was not reported. Moreover, a deep learning algorithm developed to detect the common bile duct in laparoscopic cholecystectomy reached a precision of 0.32, even with 99 procedures, 2339 frames, and annotation by experts [23]. According to our hypothesis, possible explanations for the moderate accuracy scores are the relatively small number of procedures and especially the complicated anatomy recognition tasks to detect subtle and relatively small anatomical structures.

The highest Dice coefficient (0.92) was reported by a study that trained a deep learning algorithm to detect the liver in laparoscopic cholecystectomy [10]. This study used a high number of patients (209) and training frames (2364), but did not perform any pre-training, augmentations, or pre- and post-processing. Another study that used a large number of laparoscopic cholecystectomy procedures (201) and training frames (1712) reported an IoU of 89% for segmentation of the gallbladder [27]. The deep learning algorithm used in this study was pretrained on multiple image databases. Another study presented a deep learning algorithm to segment the lungs that was trained using 25 thoracoscopic lung cancer resections and reached an IoU of 98%, which was not externally or internally validated [21]. The authors applied data augmentation to reach a total of 1890 frames. Generally, algorithms with highest accuracy used a large number of patients and frames in combination with relatively simple anatomy recognition tasks. Some of the high-accuracy algorithms used pre-training or data augmentation. The individual modeling effects of these techniques on the accuracy of the algorithm were not reported.

### Inference time

Inference times, which measures the time it takes for the algorithm to process the data and make a prediction, ranged from < 0.01 until 16 s (Table S2) [10, 28]. Six of the 13 articles (46%) published in 2020 or later reported inference times which were equal to or faster than 0.2 s; all concerned AI-based algorithms [10, 16, 18, 22, 23, 26].

## Discussion

This systematic review describes the applied methodology in building algorithms for computer-aided anatomy recognition in detail and provides a comprehensive overview of current literature on this topic. The aim of this systematic review was to gain insight into the accuracy of anatomy recognition algorithms in intrathoracic and -abdominal surgery and to identify factors that contribute to high-accuracy algorithms. Overall, accuracy of the algorithms ranged from moderate to good, and especially the recent AI algorithms reported fast inference times. However, solid comparison between studies is complicated by the wide variety of surgical procedures, anatomy recognition tasks, methodology to build algorithms, and accuracy measures of the included studies. In addition, the individual impact of different modeling steps on the algorithm accuracy was often not described. Furthermore, overall risk of bias of the included studies was high: adequate description of study populations was often missing, variation of included videos within studies was limited, and annotation of the reference standard by experts with performance validation was uncommon. Therefore, based on the reported studies, the recommendations that we listed on how to build an accurate computer-aided anatomy recognition model should be further validated. The current systematic review can be used as methodological guideline for future studies aiming to develop such algorithms.

Due to the heterogeneity among studies, it was challenging to identify factors that contribute to achieve high-accuracy algorithms. Generally, better accuracy was obtained in the studies with an AI-based approach that used datasets, which comprised a large number of video frames generated from many different surgical videos. The two included studies that used more than 200 videos to develop their AI algorithm reported IoU scores of at least 86% for detecting the liver and gallbladder [10, 27]. Machine learning algorithms will learn features that best allow them to separate the data irrespective of these features are logical, clinically relevant or a result of selection bias in the dataset. Therefore training algorithms on many different surgical videos with diversity in the assessed frames per performed surgical procedure is important, as the variation in frames makes the model more robust [12]. Most algorithms in the included studies were trained using frames annotated by expert surgeons. Two algorithms were developed using annotations by (medical) students and reached a dice coefficient of 0.70 and IoU of 74% [16, 28]. Since the performance of a data-driven algorithm is dependent on the quality of the anatomical annotation of the training data, it is highly recommended to use frames annotated or validated by surgical experts [38]. Some of the high-accuracy algorithms were developed using pre-training and data augmentation [21, 27]. As expected, recognition of small or complex anatomical structures, such as the common bile duct or specific vasculature, showed lower accuracy scores compared with larger organs, such as the liver or lung [10, 21, 23, 25].

Computer-aided anatomy recognition using surgical video analysis is an upcoming topic in research. AI-based approaches, and more specifically, deep learning algorithms, showed a vast increase in publications in the last years. These studies, using deep learning algorithms, showed promising accuracy and lower inference times compared with model-based approaches [10, 11, 16–18, 20, 22–27]. The included studies demonstrate the wide applicability of surgical algorithms, varying from organ presence recognition to classification of vascularity of organs and highlighting surgical go- and no-go zones [10, 11, 35]. This highlights the potential that computer-aided anatomy recognition may have, when applied intraoperatively.

MIS is most often technically complex and adverse surgical events remain a major issue as they are associated with morbidity and mortality, impaired recovery, prolonged hospital stay, reduced quality of life and increased costs [39, 40]. The interface between surgeon and patient, that is always present in MIS, facilitates the possible application of supporting surgical algorithms in the future. For surgeons, these algorithms may be valuable in surgical training, in improving anatomical orientation, in reducing tissue injury, and in decreasing the learning curve of novice surgeons. Moreover, algorithms indicating go- and no-go zones and safe dissection planes might be valuable to prevent damage to important structures during surgical dissection. For patients, this may result in increased safety during operations and better postoperative outcomes. The required accuracy of anatomy recognition algorithms for clinical application is still to be determined and can vary per application, depending on the specific task that needs to be completed.

The results of this systematic review are in line with a previously published systematic review on the accuracy of deep learning algorithms to analyze laparoscopic videos, which concluded that the included algorithms showed clinical potential but were limited in the quality of methodology [12]. This previous study included algorithms for prediction of operating time, surgical phase recognition, action recognition, instrument detection, and anatomy recognition, but anatomy recognition was described very briefly, detailed assessment of applied methodology was not reported, only studies investigating convolutional neural networks and deep learning were included and surgical videos of robot-assisted procedures were not assessed. The current review provides an in-depth summary on the clinical and technical possibilities (and limitations) of computer-aided anatomy recognition, and recommended standardized methodology for future studies.

For a different medical procedure, another previous systematic review and meta-analysis was published regarding the accuracy of AI for computer-aided diagnosis on colorectal polyps during colonoscopy [41]. Machine learning algorithms showed high accuracy for this task and demonstrated potential to increase adenoma detection rate. A specific AI-model for polyp detection with high accuracy used a high amount of endoscopic frames (± 5000 images) and annotation of the reference standard was performed by experienced endoscopists [42]. In this previous review, no information was provided with regards to pre-training or data augmentation. This supports the trend in our current systematic review that algorithms with good accuracy are developed using large amounts of training data and expert annotators.

To facilitate future research in anatomy recognition, larger and more diverse databases of surgical videos, labeled by surgical experts, are needed to train and test algorithms. This can only be achieved with strong collaborative effort nationally and internationally. Use of pre-training, data augmentation and external validation are known to improve the accuracy of AI-based recognition algorithms and are therefore recommended [43, 44]. In addition, we highly recommend reporting on the individual impact of such different modeling steps to demonstrate their added value in computer-aided anatomy recognition, as in all included studies in the current systematic review this detailed information is missing which complicates interpretation of individual modeling steps. The heterogeneity of accuracy measures and high risk of bias of included studies in the present systematic review highlight the need for standardization and methodological recommendations on how to build and report anatomy recognition algorithms.

Specific reporting guidelines of AI-based diagnostic algorithms are currently under development by the Standards for Reporting of Diagnostic Accuracy Study—Artificial Intelligence (STARD-AI) steering group, using a modified Delphi consensus process [45]. The STARD-AI steering group is a collective of clinicians, statisticians, epidemiologists, computer scientists, journal editors, funders, legal experts, and ethical experts. The STARD-AI guidelines will contain recommendations for developing and testing of AI-based diagnostic tests, pre-processing of data, usage of accuracy measures, explainability, and human-AI interaction. These guidelines can be applied in AI-based anatomy recognition and are expected to improve the quality and comparability of future anatomy recognition algorithms.

This systematic review has a number of limitations to consider. A meta-analysis was not possible due to the heterogeneity of the studies in outcome measures, surgical procedures, and anatomy recognition tasks. Most included algorithms were trained to identify multiple anatomical structures, so we reported on the clinically most applicable structures with the highest accuracy [10, 11, 16, 17, 23, 24, 27, 30, 32–36]. This might have resulted in a slight overestimation of the algorithm accuracies. Strong points of this systematic review include the detailed assessment of both clinical as well as technical aspects of the algorithms, the use of clinical and technical oriented search databases and the recommendations for standardized methodology on this topic. In addition, we reported on algorithms in both laparoscopic and robot-assisted surgery. The current systematic review is the first to provide an in-depth summary on anatomical recognition algorithms.

In conclusion, this systematic review describes the accuracy of computer-aided anatomy recognition in intrathoracic and—abdominal surgery. The included studies showed high overall risk of bias, especially regarding patient selection and annotation of the reference standard. The included anatomy recognition algorithms showed accuracies ranging from moderate to good. In general, high-accuracy algorithms used larger training sets, annotated by expert surgeons, simpler recognition tasks, and in some cases pre-training and data augmentation. Anatomy recognition is an upcoming field of research, but still at its infancy and not ready for clinical application yet. Larger annotated datasets and methodological improvements are required to take this research field further.

## Supplementary Information

Below is the link to the electronic supplementary material.

Supplementary file1 (DOCX 50 kb)

Supplementary file2 (DOCX 15 kb)

Supplementary file3 (DOCX 38 kb)
