# Supplementary material for: Computer-aided anatomy recognition in intrathoracic and -abdominal surgery: a systematic review
Source: Surg Endosc. 2022 Aug 4;36(12):8737–52. doi: 10.1007/s00464-022-09421-5 (PMC9652273; doi:10.1007/s00464-022-09421-5)
Supplement: Supplementary file 1 — Supplementary file1 (DOCX 50 kb) [file 464_2022_9421_MOESM1_ESM.docx]

*Supplementary document: QUADAS-2 tool for risk of bias assessment*

| **Authors** | **Risk of bias** | **Explanation** |
| --- | --- | --- |
| **Akbari et al. (2008)**[34] |  |  |
| *Patient selection* | 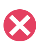 | Selection criteria for surgical videos not described. |
| *Index test* | 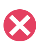 | No cross-validation or external validation. |
| *Ref. Standard* | 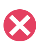 | Unclear if annotation of reference standard was performed by an expert. |
| *Flow and timing* | 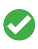 | All patients received the same reference standard and were included in analysis. |
| **Akbari et al. (2009)**[29] |  |  |
| *Patient selection* | 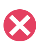 | Methods of patient selection not well-described. |
| *Index test* | 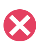 | No cross-validation or external validation. |
| *Ref. Standard* | 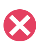 | Unclear if annotation of reference standard was performed by an expert. |
| *Flow and timing* | 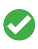 | All patients received the same reference standard and were included in analysis. |
| **Artemchuk et al. (2011)**[35] |  |  |
| *Patient selection* | 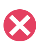 | Methods of patient selection not well-described. |
| *Index test* | 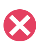 | No cross-validation or external validation. |
| *Ref. Standard* | 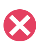 | Unclear if annotation of reference standard was performed by an expert. |
| *Flow and timing* | 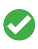 | All patients received the same reference standard and were included in analysis. |
| **Chhatkuli et al. (2014)**[31] |  |  |
| *Patient selection* | 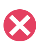 | Methods of patient selection not well-described. |
| *Index test* | 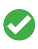 | Cross-validation or external validation used. |
| *Ref. Standard* | 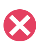 | Unclear if annotation of reference standard was performed by an expert. |
| *Flow and timing* | 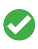 | All patients received the same reference standard and were included in analysis. |
| **Prokopetc et al. (2015)**[32] |  |  |
| *Patient selection* | 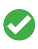 | Adequate description of study population, variation in included videos, random selection of frames. |
| *Index test* | 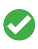 | Cross-validation or external validation used. |
| *Ref. Standard* | 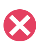 | Unclear if annotation of reference standard was performed by an expert. |
| *Flow and timing* | 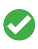 | All patients received the same reference standard and were included in analysis. |
| **Amir-Khalili et al. (2015)**[33] |  |  |
| *Patient selection* | 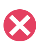 | Selection criteria for surgical videos not described. |
| *Index test* | 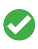 | Cross-validation or external validation used. |
| *Ref. Standard* | 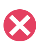 | Annotation of reference standard was performed by junior surgeon. |
| *Flow and timing* | 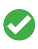 | All patients received the same reference standard and were included in analysis. |
| **Haouchine et al. (2016)**[36] |  |  |
| *Patient selection* | 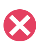 | Methods of patient selection not well-described. |
| *Index test* | 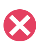 | No cross-validation or external validation. |
| *Ref. Standard* | 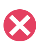 | Unclear if annotation of reference standard was performed by an expert. |
| *Flow and timing* | 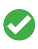 | All patients received the same reference standard and were included in analysis. |
| **Nosrati et al. (2016)**[28] |  |  |
| *Patient selection* | 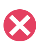 | Methods of patient selection not well-described. |
| *Index test* | 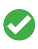 | Cross-validation or external validation used. |
| *Ref. Standard* | 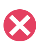 | Annotation of reference standard was performed by junior surgeon. |
| *Flow and timing* | 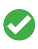 | All patients received the same reference standard and were included in analysis. |
| **Sato et al. (2019)**[30] |  |  |
| *Patient selection* | 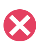 | Little variation in included surgical videos. |
| *Index test* | 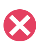 | No cross-validation or external validation used. |
| *Ref. Standard* | 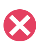 | Unclear if annotation of reference standard was performed by an expert. |
| *Flow and timing* | 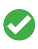 | All patients received the same reference standard and were included in analysis. |
| **Tokuyasu et al. (2020)**[23] |  |  |
| *Patient selection* | 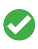 | Adequate description of study population, variation in included videos, random selection of frames. |
| *Index test* | 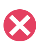 | No cross-validation or external validation. |
| *Ref. Standard* | 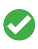 | Annotations performed by expert(s) with performance validation. |
| *Flow and timing* | 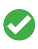 | All patients received the same reference standard and were included in analysis. |
| **Mascagni et al. (2020)**[27] |  |  |
| *Patient selection* | 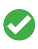 | Adequate description of study population, variation in included videos, random selection of frames. |
| *Index test* | 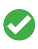 | Cross-validation or external validation used. |
| *Ref. Standard* | 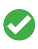 | Annotations performed by expert(s) with performance validation. |
| *Flow and timing* | 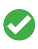 | All patients received the same reference standard and were included in analysis. |
| **Loukas et al. (2020)**[19] |  |  |
| *Patient selection* | 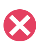 | Study population not well described. |
| *Index test* | 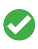 | Cross-validation or external validation used. |
| *Ref. Standard* | 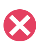 | No performance validation. |
| *Flow and timing* | 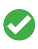 | All patients received the same reference standard and were included in analysis. |
| **Zadeh et al. (2020)**[24] |  |  |
| *Patient selection* | 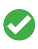 | Adequate description of study population, variation in included videos, random selection of frames. |
| *Index test* | 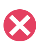 | No cross-validation or external validation. |
| *Ref. Standard* | 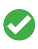 | Annotations performed by expert(s) with performance validation. |
| *Flow and timing* | 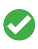 | All patients received the same reference standard and were included in analysis. |
| **Scheilkl et al. (2020)**[16] |  |  |
| *Patient selection* | 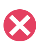 | Exclusion criteria for surgical videos not well-described. |
| *Index test* | 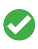 | Cross-validation or external validation used. |
| *Ref. Standard* | 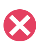 | Annotation of the reference standard by medical students. |
| *Flow and timing* | 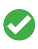 | All patients received the same reference standard and were included in analysis. |
| **Nitta et al. (2020)**[21] |  |  |
| *Patient selection* | 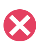 | Methods of patient selection not well-described. |
| *Index test* | 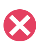 | No cross-validation or external validation. |
| *Ref. Standard* | 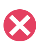 | Annotation of the reference standard by unspecified researchers. |
| *Flow and timing* | 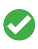 | All patients received the same reference standard and were included in analysis. |
| **Madani et al. (2020)**[10] |  |  |
| *Patient selection* | 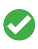 | Adequate description of study population, variation in included videos, random selection of frames. |
| *Index test* | 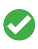 | Cross-validation or external validation used. |
| *Ref. Standard* | 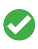 | Annotations performed by expert(s) with performance validation. |
| *Flow and timing* | 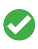 | All patients received the same reference standard and were included in analysis. |
| **François et al. (2020)**[20] |  |  |
| *Patient selection* | 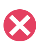 | Methods of patient selection not well-described. |
| *Index test* | 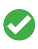 | Cross-validation or external validation used. |
| *Ref. Standard* | 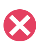 | No performance validation |
| *Flow and timing* | 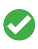 | All patients received the same reference standard and were included in analysis. |
| **Cassella et al. (2021)**[25] |  |  |
| *Patient selection* | 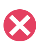 | Exclusion criteria for surgical videos not well-described. |
| *Index test* | 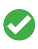 | Cross-validation or external validation used. |
| *Ref. Standard* | 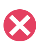 | No performance validation. |
| *Flow and timing* | 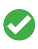 | All patients received the same reference standard and were included in analysis. |
| **Kitaguchi et al. (2021)**[22] |  |  |
| *Patient selection* | 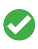 | Adequate description of study population, variation in included videos, random selection of frames. |
| *Index test* | 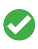 | Cross-validation or external validation used. |
| *Ref. Standard* | 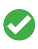 | Annotations performed by expert(s) with performance validation. |
| *Flow and timing* | 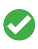 | All patients received the same reference standard and were included in analysis. |
| **Loukas et al. (2021)**[11] |  |  |
| *Patient selection* | 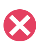 | Little variation in included surgical videos. |
| *Index test* | 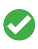 | Cross-validation or external validation used. |
| *Ref. Standard* | 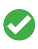 | Annotations performed by expert(s) with performance validation. |
| *Flow and timing* | 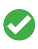 | All patients received the same reference standard and were included in analysis. |
| **Caballas et al. (2021)**[26] |  |  |
| *Patient selection* | 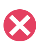 | Little variation in included surgical videos. Only one included patient. |
| *Index test* | 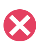 | No cross-validation or external validation. |
| *Ref. Standard* | 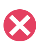 | Unclear if annotation of reference standard was performed by an expert. |
| *Flow and timing* | 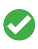 | All patients received the same reference standard and were included in analysis. |
| **Bamba et al. (2021)**[17] |  |  |
| *Patient selection* | 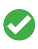 | Adequate description of study population, variation in included videos, random selection of frames. |
| *Index test* | 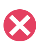 | No cross-validation or external validation. |
| *Ref. Standard* | 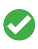 | Annotations performed by expert(s) with performance validation. |
| *Flow and timing* | 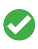 | All patients received the same reference standard and were included in analysis. |
| **Kumazu et al. (2021)**[18] |  |  |
| *Patient selection* | 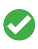 | Adequate description of study population, variation in included videos, random selection of frames. |
| *Index test* | 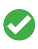 | Cross-validation or external validation used. |
| *Ref. Standard* | 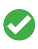 | Annotations performed by expert(s) with performance validation. |
| *Flow and timing* | 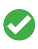 | All patients received the same reference standard and were included in analysis. |
