# Supplementary material for: Computer-aided anatomy recognition in intrathoracic and -abdominal surgery: a systematic review
Source: Surg Endosc. 2022 Aug 4;36(12):8737–52. doi: 10.1007/s00464-022-09421-5 (PMC9652273; doi:10.1007/s00464-022-09421-5)
Supplement: Supplementary file 2 — Supplementary file2 (DOCX 15 kb) [file 464_2022_9421_MOESM2_ESM.docx]

Table S1. Explanation of performance measures

| Performance measure | Formula | Range | Definition |
| --- | --- | --- | --- |
| Dice/F1 score | $Dice=\frac{2\times TP}{2x TP+FP+FN}$ | 0-1 | Measures the overlap between two segmentations; similar to IoU. |
| IoU/Jaccard index | $IoU=\frac{TP}{TP+FP+FN}$ | 0-100% | Measures the overlap between two segmentations; similar to Dice.. |
| Accuracy | $Accuracy= \frac{TP+TN}{TP+FP+FN+TN}$ | 0-1 | The proportion of correct predictions (both positive and negative). |
| Precision | $Precision=\frac{TP}{TP+FP}$ | 0-1 | The fraction of positive predictions that are true positive. |
| ROC | $x=1-specificity$    $y=sensitvity$ | N/A | A graphic representation showing true positive rate (TPR) against the false positive rate (FPR). |
| AUC | $AUC= \int TPR d\left( FRP \right)$ | 0-1 | The area under the ROC curve. Often used to compare predictive models. |
| FPR | $FPR=\frac{FP}{FP+TN}$ | 0-1 | The rate by which the algorithm predicts positive, while actually negative. |
| FNR | $FPR=\frac{FN}{FN+TP}$ | 0-1 | The rate by which algorithm predicts negative, while actually positive. |
| Sensitivity/Recall | $Sensitivity=\frac{TP}{TP+FN}$ | 0-1 | The ability to detect a true positive. |
| Specificity | $Specificity=\frac{TN}{TN+FP}$ | 0-1 | The ability to detect a true negative. |
| One-error | $1-err(f,x,Y) = \left\{ \begin{aligned} 0 if {argmax}_{k} f_{k}(x) \epsilon Y \\ 1 otherwise \end{aligned} \right.$ | 0-1 | Proportion of bags in which the label with the highest probability is misclassified. |
| Ranking-loss | $RL(f,x,Y) = \frac{1}{\vert Y\vert\vert Y\vert}\vert(i,j) \in Y x Y s.t. r_{i}(x)\leq r_{j}(x)\vert$ | 0-1 | The average fraction of pairs that are not correctly labeled. |
| Hamming-loss | $L_{Hamming(y,\hat{y}) = \frac{1}{n_{Labels}}\sum_{j=0}^{n_{labels-1}} 1(\hat{y_{j}}\neq y_{j})}$ | 0-1 | Proportion of all misclassified labels. |
| Coverage | $coverage(y,\hat{f}) = \frac{1}{n_{samples}}*\sum_{n=0}^{n_{samples-1}} {max}_{j:yij}=1 {rank}_{ij}$ | 0-1 | Proportion of the ranking distances of misclassified labels with the minimum probability. |
| mAP | $mAP= \frac{1}{N}\sum_{i=1}^{N} {AP}_{i}$ | 0-1 | Mean area under the precision-recall curve. |

AP: Average Precision; AUC: Area Under the Receiving Operating Characteristic Curve; IoU: Intersection over Union; FNR: False Negative Rate; FPR: False Positive Rate; mAP: Mean Average Precision; N: Number of cases; N/A; Not Applicable; ROC: Receiving Operating Charateristic
