# Supplementary material for: Computer-aided anatomy recognition in intrathoracic and -abdominal surgery: a systematic review
Source: Surg Endosc. 2022 Aug 4;36(12):8737–52. doi: 10.1007/s00464-022-09421-5 (PMC9652273; doi:10.1007/s00464-022-09421-5)
Supplement: Supplementary file 3 — Supplementary file3 (DOCX 38 kb) [file 464_2022_9421_MOESM3_ESM.docx]

*Table S2: Technical details of the included algorithms*

| **Author (year)** | **Surgical procedure** | **Pre-training** | **Data augmentation** | **External validation** | **Validation strategy** | **Inference time** | **Accuracy** | | |
| --- | --- | --- | --- | --- | --- | --- | --- | --- | --- |
|  |  |  |  |  |  |  | **Dice** | **IoU** | **Other** |
| Akbari et al. (2008)[34] | Abdominal laparoscopy | N/A | N/A | No | Clinical testing | 3-4 seconds | NR | NR | Sensitivity: 0.95  Specificity: 0.92 |
| Akbari et al. (2009)[29] | Lap. cholecystectomy | N/A | N/A | No | Clinical testing | 4-5 seconds | NR | NR | Sensitivity: 0.95  Specificity 0.96 |
| Artemchuk et al. (2010)[35] | Abdominal laparoscopy | N/A | N/A | No | NR | NR | NR | NR | Precision: 0.86 |
| Chhatkuli et al. (2014)[31] | Lap. gynaecological procedure | N/A | N/A | No | NR | NR | 0.80 | NR | NR |
| Prokopetc et al. (2015)[32] | Lap. gynaecological procedure | N/A | N/A | No | 7 fold cross validation | NR | NR | NR | FPR: 0.21  Recall: 0.95 |
| Amir-Khalili et al. (2015)[33] | Robot-assisted nephrectomy | N/A | N/A | No | Clinical cases | NR | 0.50 (SD: 0.24) | NR | AUC: 0.72 (SD: 0.05) |
| Haouchine et al. (2016)[36] | Robot-assisted hepatectomy | N/A | N/A | No | Clinical cases | NR | 0.81 | NR | NR |
| Nosrati et al. (2016)[28] | Robot-assisted nephrectomy | N/A | N/A | No | Clinical cases | 16 seconds | 0.70 | NR | Accuracy: 0.88  FPR: 0.07 |
| Sato et al. (2019)[30] | Lap. hysterectomy | N/A | N/A | No | NR | NR | NR | NR | NR |
| Nitta et al. (2020)[21] | Thoracoscopic lung cancer resection | N/A | Parallel movement, affine transformation, zooming, contrast changes | No | N/A | NR | NR | 98% | NR |
| Tokuyasu et al. (2020)[23] | Lap. cholecystectomy | N/A | Rotation, shearing, zoom, contrast changes | No | N/A | 0.03 seconds | NR | NR | Precision: 0.32 |
| Mascagni et al. (2020)[27] | Lap. cholecystectomy | ImageNet + Microsoft COCO + PASCAL VOC | N/A | No | 5-fold cross validation | NR | NR | 89% (SD: 0.9) | NR |
| Loukas et al. (2020)[19] | Lap. cholecystectomy | N/A | N/A | No | 10-fold cross validation | <0.01 seconds | NR | NR | Accuracy: 0.79 (SD: 0.02) |
| Zadeh et al. (2020)[24] | Lap. hysterectomy | ImageNet | N/A | No | NR | NR | NR | 85% | Recall: 0.97  Precision: 0.99 |
| Sheilkl et al. (2020)[16] | Lap. cholecystectomy | ImageNet | Gaussian blurs, rotations, horizontal flips, and combinations | No | Uniform random sampling | <0.04 seconds | NR | 74% | NR |
| Madani et al. (2020)[10] | Lap. cholecystectomy | N/A | N/A | Yes | 10-fold cross-validation | <0.01 seconds | 0.92 (SD: 0.10) | 86% (SD:12) | Sensitivity: 0.93 (0.10) specificity: 0.96 (0.04) |
| François et al. (2020)[20] | Lap. gynaecological procedure | Semantic boundary dataset | N/A | No | NR | NR | NR | NR | Reprojection error: 62.44 pixels |
| Casella et al. (2021)[25] | Robot-assisted nephrectomy | N/A | Random set of flipping, zooming, and shearing | No | Holdout cross validation | NR | 0.72 (SE: 0.09) | NR | Recall: 0.51(SE: 0.14)  Precision: 0.90 (SE: 0.11) |
| Kitaguchi et al. (2021)[22] | Lap. TATME | ImageNet | Horizontal flip | No | 5-fold cross validation | 0.09 seconds | 0.71 (SD: 0.04) | NR | NR |
| Loukas et al. (2021)[11] | Lap. cholecystectomy | N/A | Horizontal flip, vertical flip, and rotation | No | 5-fold cross validation | NR | NR | NR | AUC: 0.95  Precision: 0.91 |
| Caballas et al. (2021)[26] | Lap. cholecystectomy | Yes (not specified) | N/A | No | N/A | 0.05 seconds | NR | NR | Average precision: 0.89 |
| Bamba et al. (2021)[17] | Abdominal laparoscopy | N/A | N/A | No | N/A | NR | NR | NR | Precision 0.91 (95% CI: 0.88 -0.94)  Recall: 0.93 (95% CI 0.90 – 0.96) |
| Kumazu et al. (2021)[18] | Robot-assisted gastrectomy | N/A | N/A | No | Questionnaires on sensitivity and misrecognition | 0.2 seconds | 0.55 | NR | Recall: 0.61 |

AUC: Area Under receiver operating characteristic Curve; COCO: Common Objects in Context; Lap.: laparoscopic; N/A; Not Applicable; NR: Not Reported; SD: Standard Deviation; SE: Standard Error; TATME; Transanal Total Mesorectal Excision; VOC; Virtual Object
